# Data to genetic risk assessment on high-density cholesterol level associated polymorphisms in Hungarian general and Roma populations

**DOI:** 10.1016/j.dib.2017.07.053

**Published:** 2017-07-26

**Authors:** Péter Pikó, Szilvia Fiatal, Zsigmond Kósa, János Sándor, Róza Ádány

**Affiliations:** a MTA-DE Public Health Research Group of the Hungarian Academy of Sciences, Faculty of Public Health, University of Debrecen, Debrecen 4028, Hungary; b Department of Preventive Medicine, Faculty of Public Health, University of Debrecen, Debrecen 4028, Hungary; c WHO Collaborating Centre on Vulnerability and Health, Department of Preventive Medicine, Faculty of Public Health, University of Debrecen, Debrecen 4028, Hungary; d Department of Health Visitor Methodology and Public Health, Faculty of Health, University of Debrecen, Nyíregyháza 4400, Hungary

**Keywords:** Single nucleotide polymorphism, Genetic susceptibility, Genetic risk score, High-density lipoprotein cholesterol, Roma population

## Abstract

Data obtained by genotyping single nucleotide polymorphisms (SNPs) related to high-density lipoprotein cholesterol (HDL-C) levels were utilized in Genetic Risk Score [unweighted (GRS) and weighted (wGRS)] computation on Hungarian general and Roma populations. The selection process of the SNPs as well as the results obtained are published in our research article (Piko et al., 2017) [1]. Linkage analyses were performed by study groups. Study populations were stratified by quintiles of weighted Genetic Risk Score. Multivariate linear regression analyses were performed using Genetic Risk Scores and HDL-C levels as dependent variables; and ethnicity, sex and age as independent variables. The study subjects were categorized into quintiles according their wGRS values. Associations of Genetic Risk Scores with plasma HDL-C levels (as a continuous variable) were observed in both populations. Finally, the two populations were merged and analyzed together by multivariate logistic regression where reduced plasma HDL-C level was the dependent variable; while ethnicity, age and sex were the independent ones.

**Specifications Table**

**Table:** 

Subject area	*Biology*
More specific subject area	*Molecular genetics, Public health genomics*
Type of data	*Figure, Table*
How data was acquired	*Survey, Blood sample collection, MassARRAY platform (Sequenom Inc., San Diego, CA, USA) with iPLEX Gold chemistry*
Data format	*Analyzed*
Experimental factors	*Genomic DNA from peripheral blood was isolated*
Experimental features	*Genotyping method of SNPs was based on MALDI-TOF (Matrix Assisted Laser Desorption-Ionisation-Time Of Flight) analysis, performed on MassARRAY Platform.*
Data source location	*Debrecen, Hungary, Latitude:* 47.544062, 21° 38′ 25′′ E *& Longitude:* 21.64283, 47° 32′ 33′′ N
Data accessibility	*Data are presented in this article; DNA sample and raw data are available for further analyses in collaborative studies*

**Value of the data**

• Several studies describe the health status of the Roma, which constitutes the largest ethnic minority in Europe however studies focusing on their genetic predisposition to common chronic diseases are scarce.
• Genetic background of atherosclerosis among Roma as well as the general Hungarian population can be studied separately or in international cohort.
• Genetic risk score assessment can be further utilized to compare susceptibility to reduced HDL-C level among different population groups.

## 1 Data

Distribution of SNPs related to HDL-C level were analysed in the Hungarian Roma and general populations and weighted Genetic Risk Scores were defined and used to categorize the population into quintiles.

## 2 Experimental design, materials and methods

### 2.1 Subjects

Study involved subjects of samples investigated during recent cross sectional surveys [2], [3]. The Roma sample is representative to the Roma population living settlements in North-East Hungary in terms of age and sex and includes 646 individuals (Roma). The “General” sample consisting of 1542 individuals representative for the Hungarian general population in terms of geographic, age and sex distributions.

### 2.2 DNA extraction

DNA was isolated using a MagNA Pure LC system (Roche Diagnostics, Basel, Switzerland) with a MagNA Pure LC DNA Isolation Kit–Large Volume according to the manufacturer's instructions. Extracted DNA was eluted in 200 µl MagNA Pure LC DNA Isolation Kit-Large Volume elution buffer.

### 2.3 SNP selection

**Fig. 1:**
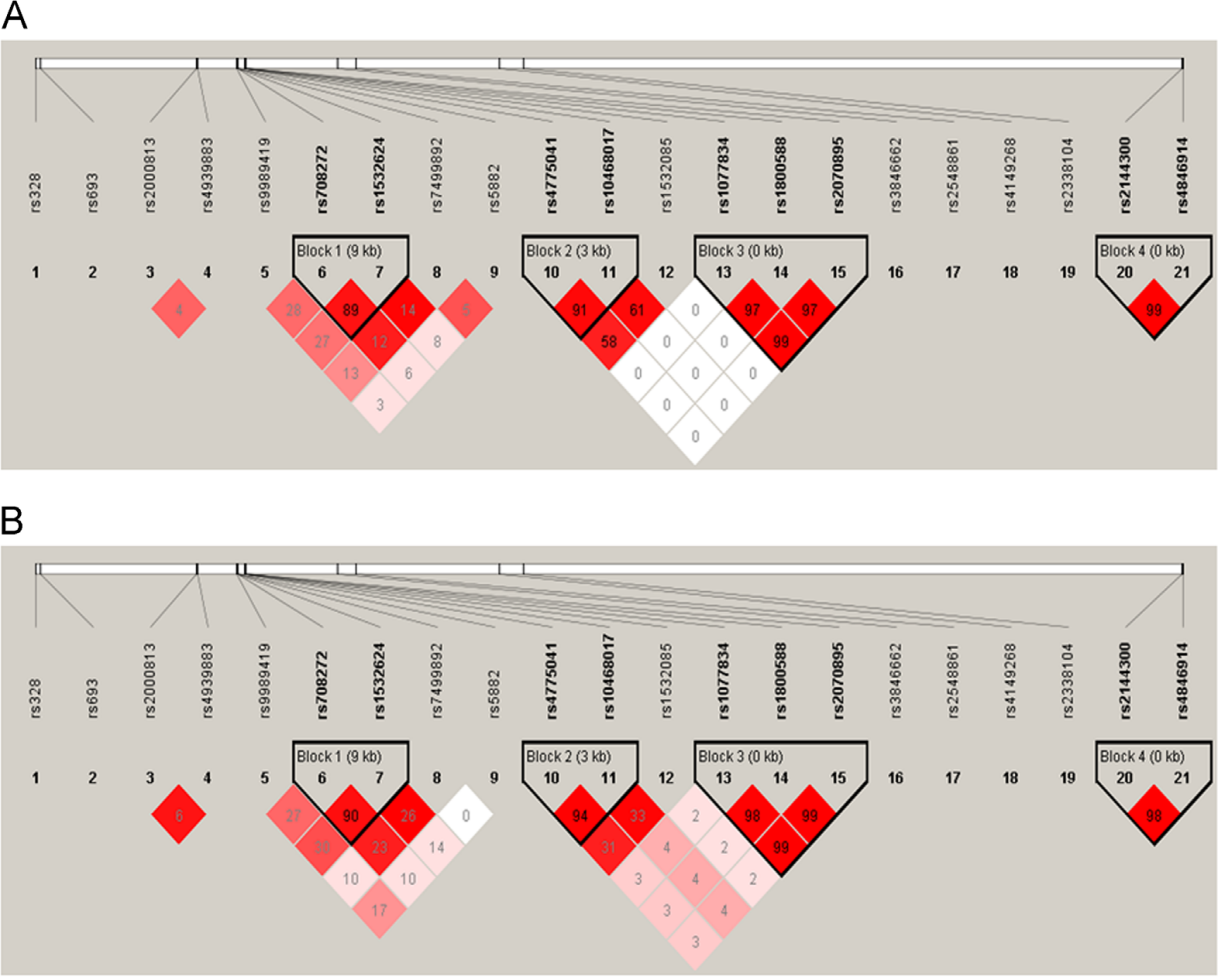
Haplotype block organization of SNPs related to high-density lipoprotein cholesterol level on the LD maps for the Hungarian general (A) and Roma (B) populations. Linkage analyses were performed separately in the study populations. According to the LD map generated by Haploview, there are four haplotype blocks (outlined in a bold black line) consisting of variants that are in high LD. The blocks were formed by the SNPs of the *CETP*, *LIPC* and *GALNT2* genes. The numbers above the map show the rs numbers of SNPs. The colour scheme is a standard Haploview colour scheme (white *D*′<1 and LOD<2, shades of pink/red: *D*′<1 and LOD≥2, and bright red *D*′=1 and LOD≥2). Numbers in squares are *D*′ values.

A systematic literature review on the PubMed, HuGE Navigator and Ensembl databases was conducted to identify SNPs most strongly associated with HDL-C metabolism (Table 1 and Fig. 1). The selection process of the SNPs is demonstrated in detail in our research article [1].

**Table 1:** List of the SNPs which were involved in the research.

Nearest Gene	Gene (short)	SNP (rs number)	Chromosome
Apolipoprotein B	APOB	rs693	2
ATP-binding cassette transporter ABCA1	ABCA1	rs4149268	9
Cholesteryl ester transfer protein	CETP	rs1532624	16
Cholesteryl ester transfer protein	CETP	rs5882	16
Cholesteryl ester transfer protein	CETP	rs708272	16
Cholesteryl ester transfer protein	CETP	rs7499892	16
Cholesteryl ester transfer protein	CETP	rs9989419	16
Endothelial lipase	LIPG	rs2000813	18
Endothelial lipase	LIPG	rs4939883	18
Hepatic lipase	LIPC	rs10468017	15
Hepatic lipase	LIPC	rs1077834	15
Hepatic lipase	LIPC	rs1532085	15
Hepatic lipase	LIPC	rs1800588	15
Hepatic lipase	LIPC	rs2070895	15
Hepatic lipase	LIPC	rs4775041	15
HMG-CoA Reductase	HMGCR	rs3846662	5
Lipoprotein lipase	LPL	rs328	8
Polypeptide N-acetylgalactosaminyltransferase 2	GALNT2	rs2144300	1
Polypeptide N-acetylgalactosaminyltransferase 2	GALNT2	rs4846914	1
Potassium channel tetramerization domain containing 10	KCTD10	rs2338104	12
WW Domain Containing Oxidoreductase	WWOX	rs2548861	16

### 2.4 Genotyping

Genotyping was performed on a MassARRAY platform (Sequenom Inc., San Diego, CA, USA) with iPLEX Gold chemistry. Validation, concordance analysis and quality control were conducted by the facility according to their protocols.

### 2.5 Statistical analyses

Two-sided t tests were used to compare the distribution of genetic risk scores in populations. To reveal the association between genetic risk, serum HDL-C level and ethnicity several statistical models were used (Table 1, Table 2, Table 3, Table 4, Table 5, Table 6).

**Table 2:** Distribution of study populations by wGRS quintiles.

	**Hungarian general population (%)**	**Hungarian Roma population (%)**	***p-value***
**1**st **quintile of wGRS (0.15–≤0.30)**	1.83	0.51	0.025
**2**nd **quintile of wGRS (0.31–≤0.45)**	17.18	10.45	<0.001
**3**rd **quintile of wGRS (0.46–<0.59)**	48.38	49.14	0.756
**4**th **quintile of wGRS (0.6–≤ 0.74)**	30	34.76	0.037
**5**th **quintile of wGRS (0.75–0.88)**	2.61	5.14	0.004

**Table 3:** Output of multiple regression models using unweighted and weighted genetic risk scores as dependent variable and ethnicity, age and sex as independent variables.

Dependent variable: GRS		R Square=0.009	
**Independent variables**	**Coefficient**	***p-value***	***β***
**Ethnicity (Roma vs. General)**	0.667	<0.001	0.092
**Sex women vs. men)**	0.106	0.477	0.016
**Age**	−0.0003	0.068	−0.001
*β*: relative strength of predictors			
Dependent variable: wGRS		R Square=0.017	
**Independent variables**	**Coefficient**	***p-value***	***β***
**Ethnicity (Roma vs. General)**	0.029	<0.001	0.125
**Sex (women vs. men)**	−0.001	0.774	−0.006
**Age**	−0.0002	0.202	−0.028
*β*: relative strength of predictors			

Multivariate regression analysis using age, sex as covariates did not change the inference neither for the GRS nor for wGRS.

**Table 4:** Proportion of subjects with reduced plasma HDL-C level in the General and Roma populations according to wGRS quintiles.

	**1st quintile of wGRS (0.15–≤0.30)**	**2nd quintile of wGRS (0.31–≤0.45)**	**3rd quintile of wGRS 0.46–<0.59)**	**4th quintile of wGRS**	**5th quintile of wGRS (0.75–0.88)**	***p-values*****for trend**
				**(0.6–≤0.74)**		
**General (Men; Women)**	***N*=26 (8;18)**	***N*=241 (115;126)**	***N*=681 (320;361)**	***N*=417 (200;217)**	***N*=36 (21;15)**	
Average HDL-C level (mmol/l)	1.56	1.47	1.41	1.38	1.33	0.021
Reduced plasma HDL-C (%)	11.54	23.77	27.8	28.5	31.43	0.083
**Roma (Men; Women)**	***N*=3 (1;2)**	***N*=61 (28;33)**	***N*=287 (112;175)**	***N*=203 (76;127)**	***N*=30 (11;19)**	
Average HDL-C level (mmol/l)	1.26	1.24	1.23	1.2	1.09	0.076
Reduced plasma HDL-C (%)	33.33	44.26	49.83	52.22	56.67	0.054

**Table 5:** Association of GRSs with plasma HDL-C^a^ level by study groups.

	**Hungarian General**		**Hungarian Roma**	
	***β* (95% CI)**	***p-value***	***β* (95% CI)**	***p-value***
**GRS**				
**Model I**	−0.01 (−0.018 to −0.003)	0.004	−0.013 (−0.023 to −0.003)	0.011
**Model II**	−0.011 (−0.018 to −0.004)	0.003	−0.013 (−0.023 to −0.003)	0.009
**wGRS**				
**Model III**	−0.243 (−0.466 to −0.020)	0.033	−0.318 (−0.633 to −0.002)	0.049
**Model IV**	−0.205 (−0.420 to 0.101)	0.062	−0.336 (−0.651 to −0.21)	0.036

The association of GRS and wGRS with plasma HDL-C level were evaluated under unadjusted regression models (Model I and III) and under regression models adjusted for age and sex (Model II and IV) separately in Roma and general subjects. In all models the HDL-C was the dependent variable, the GRS/wGRS were the independent variables.

95% CI: 95% confidence interval

a HDL-C values were non-normally distributed and were transformed using a two-step approach suggested by Templeton [4].

**Table 6:** The association of HDL-C level with genetic risk scores adjusted by ethnicity, sex and age.

Dependent variable: reduced plasma HDL-C level		R Square=0.046
**Independent variables**	**OR (95% CI)**	***p-value***
**Genetic constitution defined by GRS**	1.07 (1.04–3.31)	<0.001
**Ethnicity (Roma vs. General)**	2.70 (2.19–3.31)	<0.001
**Sex (women vs. men)**	0.99 (0.81–1.20)	0.942
**Age**	1.00 (0.99–1.01)	0.393
Dependent variable: reduced plasma HDL-C level		R Square=0.042
**Independent variables**	**OR (95% CI)**	***p-value***
**Genetic constitution defined by wGRS**	3.89 (1.56–9.69)	0.004
**Ethnicity (Roma vs. General)**	2.69 (2.19–3.31)	<0.001
**Sex (women vs. men)**	1.00 (0.83–1.21)	0.993
**Age**	1.00 (0.99–1.01)	0.353

OR: odds ratio.

## Ethical approval

All procedures performed in studies involving human participants were in accordance with the ethical standards of the institutional and/or national research committee and with the 1964 Helsinki declaration and its later amendments or comparable ethical standards.

This study was approved by the Ethical Committee of the University of Debrecen, Medical Health Sciences Centre (Reference no. 2462-2006) and by the Ethical Committee of the Hungarian Scientific Council on Health (Reference nos. NKFP/1/0003/2005 and 8907-O/2011-EKU).

This article does not contain any studies with animals performed by any of the authors.
