# Supplementary material for: Data to genetic risk assessment on high-density cholesterol level associated polymorphisms in Hungarian general and Roma populations
Source: Data Brief. 2017 Jul 26;14:354–9. doi: 10.1016/j.dib.2017.07.053 (PMC5545818; doi:10.1016/j.dib.2017.07.053)
Supplement: Supplementary file 2 — Supplementary material [file mmc2.docx]

**Supplementary material**

- *Figure 1* depicts the haplotype block organization of SNPs related to HDL-C level on LD maps for the Hungarian General and Roma populations.
- *Table 1* list of the SNPs which were involved in the research
- *Table 2* describes the distribution of study populations by wGRS quintile.
- *Table 3* shows the output of multiple regression models using unweighted and weighted genetic risk scores as dependent variable and ethnicity, age and sex as independent variables.
- *Table 4* summarizes the proportion of subjects with reduced plasma HDL-C levels in the General and Roma populations according to wGRS quintiles.
- *Table 5* illustrates the association of genetic risk scores with plasma HDL-C level by study populations.
- *Table 6* shows the association of HDL-C level with genetic risk scores adjusted by ethnicity, sex, age covariates using multiple logistic regression.
